# Time trend prediction of multidrug-resistant/rifampicin-resistant tuberculosis in treatment initiation centers of North East Ethiopia (2015–2023)

**DOI:** 10.1186/s12879-025-11816-3

**Published:** 2025-10-21

**Authors:** Enkuneh Atnafu Abegaz, Metadel Adane Mesfin, Getaw Walle Bazie

**Affiliations:** 1 College of Medicine and Health Sciences, School of Public Health, Department of Epidemiology and Biostatistics, Wollo University, Dessie, Ethiopia; 2 College of Medicine and Health Sciences, Department of Environmental Health Science, Wollo University, Dessie, Ethiopia

**Keywords:** Multidrug/rifampicin-resistant tuberculosis, Time series analysis, Seasonal autoregressive integrated moving average, Treatment initiation centers

## Abstract

**Background:**

Multidrug-resistant and rifampicin-resistant tuberculosis (MDR/RR-TB) represents a major public health threat and a significant obstacle to global TB control. Analysing trends and forecasting future patterns is critical for effective resource planning. However, the application of predictive modelling for MDR/RR-TB has not been widely explored in Ethiopia.

**Objective:**

This study aimed to analyse the temporal trends and develop a forecasting model for MDR/RR-TB cases recorded at treatment initiation centres in Northeast Ethiopia between 2015 and 2023.

**Methods:**

A retrospective study of all MDR/RR-TB cases diagnosed from January 2015 to December 2023 in Northeast Ethiopia was conducted using data retrieved from six treatment initiation centers (TIC) registries. Data were collected via Kobo Toolbox and analysed with SPSS v27 for descriptive statistics. Seasonal ARIMA models were developed in R to assess trends and generate forecasts, with model selection based on AIC, BIC, and residual diagnostics. Data quality was ensured through verification and consistency checks.

**Results:**

From an initial 409 identified individuals, 372 were included in the final analysis after excluding transferred cases. Annual case counts demonstrated instability, with a notable rise between 2017 and 2019 (up to 63.6%) and a distinct decline during 2020–2021, followed by a sharp increase in early 2022. A clear seasonal pattern was observed, with case troughs occurring in August and peaks during the dry season (Bega), followed by a decline in December.

**Conclusion:**

MDR/RR-TB case trends in Northeast Ethiopia exhibited significant fluctuations over the study period. The pronounced decline in 2020–2021 was likely attributable to service disruptions from the COVID-19 pandemic and regional conflict, while the subsequent surge may reflect a recovery of case detection efforts and the conflict’s impact on transmission. TB control programs should prioritize high-risk seasonal periods and ensure resilient systems for timely diagnosis and treatment access amidst external shocks.

## Introduction

Tuberculosis (TB) strains that develop resistance to anti-TB drugs are significantly more difficult to treat than drug-susceptible strains and pose a substantial challenge to patients, healthcare workers, and health systems [1, 2]. Multidrug-resistant TB (MDR/RR-TB) is TB that does not respond to at least isoniazid and rifampicin, the two most powerful anti-TB drugs, or to rifampicin alone [3]. Drug-resistant TB is equally infectious and transmissible as drug-susceptible TB; however, delays in detecting resistance and prolonged infectious periods increase the likelihood of transmission and the emergence of additional resistance [4].

Clinical management of MDR/RR-TB is complex. Guidelines recommend various drug combinations and treatment durations, but no single standardized regimen exists. As a result, treatment often requires the expertise of multidisciplinary teams (“TB Consilium”), which are not widely accessible. This lack of specialized support complicates efforts to decentralize MDR/RR-TB care [5].

In 2023, approximately 400,000 individuals worldwide developed multidrug- or rifampicin-resistant tuberculosis (MDR/RR-TB), yet only 44% were diagnosed and initiated treatment. Despite a 68% treatment success rate amongst those treated in 2021 [6], these statistics highlight persistent gaps in global MDR/RR-TB care, underscoring the urgent need for health authorities to enhance disease control and management strategies to curb its spread and prevent subsequent mortality [7, 8].

In Ethiopia, MDR/RR-TB remains a pressing public health concern [9]. To strengthen the response, several initiatives have been introduced. GeneXpert, a rapid molecular diagnostic tool, has improved early and accurate detection, while national guidelines emphasize second-line drug regimens and patient-centered strategies such as community-based treatment and psychosocial support. These approaches aim to improve adherence, treatment outcomes, and reduce transmission of resistant strains [10]. Given Ethiopia’s status as one of the 30 high-burden countries for TB, MDR-TB, and TB/HIV co-infection, it is essential to closely monitor the epidemic. Understanding recent trends in MDR/RR-TB in regions such as Amhara is critical for informing prevention and control strategies [11].

Monitoring drug resistance also guides TB control policies, as managing resistant strains requires second-line drugs that are more toxic, costly, and less effective than standard first-line regimens [12]. Direct medical expense of patients with MDR/RR-TB was still significant, and domestic migrants, hospitalization, long treatment duration, and high health insurance rates increased the financial burden on MDR/RR-TB patients [13].

Time series analysis is a vital statistical method in public health for evaluating temporal data. It employs models such as ARIMA and seasonal decomposition to identify trends, seasonal patterns, and outliers in disease incidence and other health metrics. These insights support evidence-based interventions and enhance population health strategies [14].

Although Ethiopia has developed national guidelines and several studies have explored the epidemiology and risk factors of MDR/RR-TB, A clear gap in the existing literature is the limited evidence on long-term time-trend predictions of MDR/RR-TB specifically in Northeast Ethiopia, where localized forecasting studies are scarce. This study assessed the trends of MDR/RR-TB cases and projected the likely future burden over the next decade. The findings provide critical input for policymakers and TB program implementers by indicating whether cases are expected to decline, remain stable, or increase. Ultimately, the study contributes to national and global efforts to end TB by 2030 through evidence-based forecasting and control strategies.

## Methods

### Study area and period

**Fig. 1:**
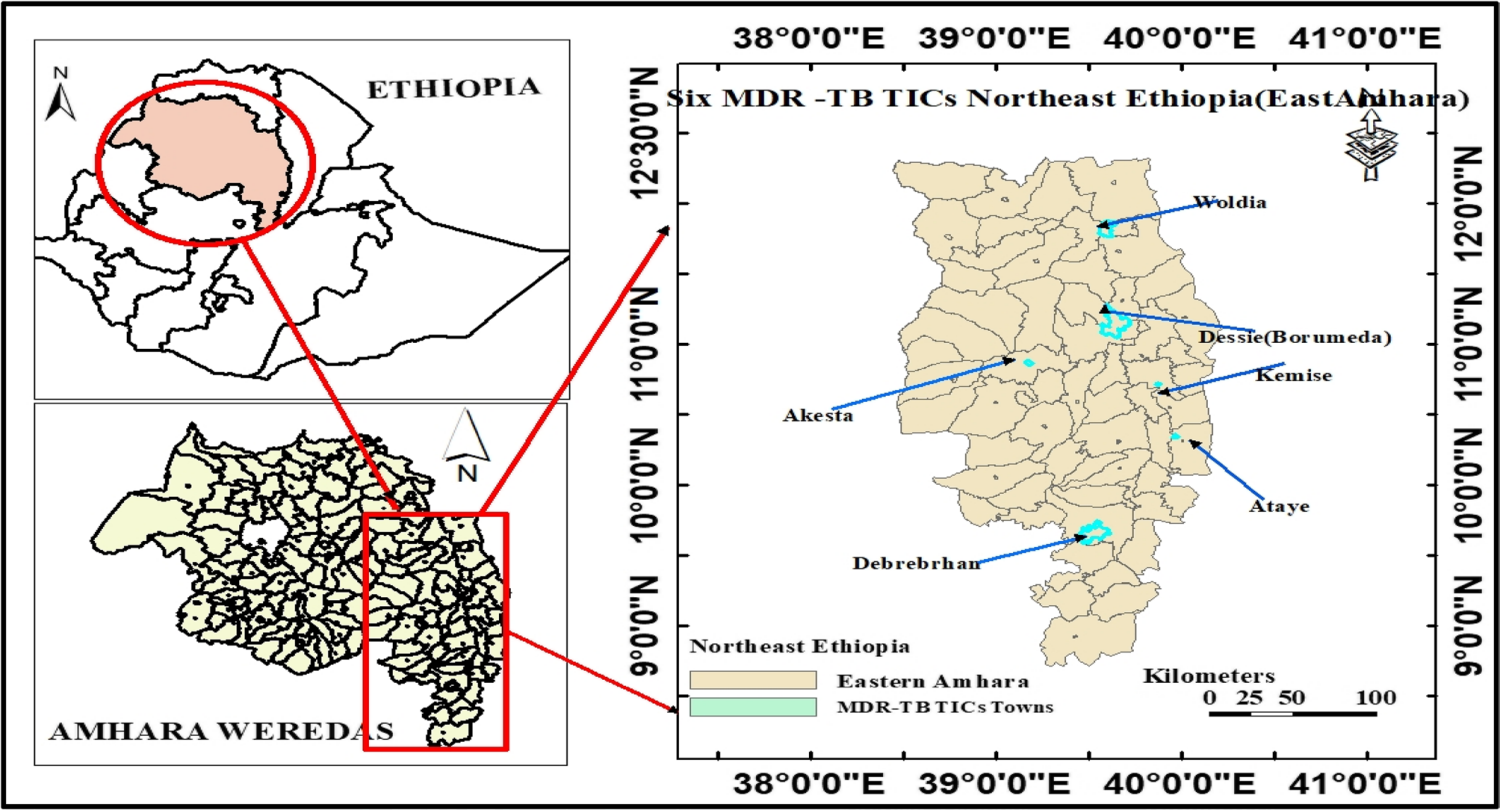
Study area map showing the MDR/RR TICs of Northeast Ethiopia (East Amhara)

The study was conducted in Northeast Ethiopia, specifically in the Eastern Amhara region, which comprises five administrative zones. According to the Ethiopian Statistical Service, the projected population of this area in 2024 was 9,124,592, of whom 4,564,372 (50.02%) were female [15]. Six MDR-TB treatment centers are located in the study area: Woldia Comprehensive Specialized Hospital, Boru-Meda General Hospital, Ataye District Hospital, Debre-Berhan Comprehensive Specialized Hospital, Kemise General Hospital, and Akesta General Hospital. All MDR-TB Treatment Initiating Centers (TICs) in the region were included in the study [16, 17] (Fig. 1). Data for this research were collected from September 30, 2024, to October 30, 2024.

### Data source

The primary data for this study were obtained from the drug-resistant tuberculosis (DR-TB) registries of six Treatment Initiation Centers (TICs) in Northeast Ethiopia. Aggregated records of MDR/RR-TB cases diagnosed between January 1, 2015, and December 31, 2023, were extracted for analysis.

### Population

The study population comprised all patients diagnosed with MDR/RR-TB at the six Treatment Initiation Centers in Northeast Ethiopia between 2015 and 2023, with data collected retrospectively.

### Inclusion criteria

Patients of all ages diagnosed with MDR/RR-TB using standard diagnostic methods (culture, DST, or molecular tests) between 2015 and 2023, who initiated treatment at any of the six designated centers in Northeast Ethiopia and had complete medical records available for analysis.

### Exclusion criteria

#### Transfer cases

Patients who were transferred to or from other treatment centers during their course of therapy were excluded due to incomplete treatment records at the study sites.

### Study variables

Key variables included year (2015–2023) and month to assess seasonality, along with demographic and clinical factors such as age, sex, diagnostic method, treatment regimen, prior TB history, disease site, resistance type, HIV status, nutritional status, and treatment outcomes.

### Operational definitions

#### Multi-drug resistant tuberculosis

Defined as tuberculosis caused by *Mycobacterium tuberculosis* strains resistant to at least the two most powerful first-line anti-TB drugs, isoniazid and rifampicin [18].

#### Rifampicin resistant

Tuberculosis caused by *Mycobacterium tuberculosis* strains that are resistant to rifampicin, with or without resistance to other anti-TB drugs [19].

Rifampicin-resistant TB (RR-TB) strains are those that are not susceptible to rifampicin on the basis of DST and, as a result, are eligible for treatment with MDR-TB regimens. Rifampicin-resistant TB strains may be susceptible or resistant to isoniazid (i.e. MDR-TB), or resistant to other first-line TB medicines (polyresistant) or second-line TB medicines (e.g. extensively drug-resistant [XDR]-TB).MDR-TB and RR-TB cases are often grouped as MDR/RR-TB [20].

#### Incidence of MDR-TB

The number of new and relapse MDR TB cases diagnosed annually from 2015 to 2023.

### Data collection procedures

Data for this study were obtained from DR-TB registries and the District Health Information Software 2 (DHIS2), with support from two trained health professionals to ensure accuracy. Temporal and demographic variables were collected to analyze trends and characterize the affected population. Information from six TICs was gathered using Kobo Toolbox with a structured questionnaire administered on mobile devices and computers, both online and offline, to ensure accessibility.

### Data quality assurance

Quality assurance in data collection using Kobo Toolbox and exporting to SPSS was conducted through automated checks in Kobo Toolbox to identify missing or inconsistent responses, data completeness monitoring, manual cross-checking of collected data against source documents, and duplicate entry verification.

### Data analysis

#### Descriptive analysis

Data collected using the ODK Toolbox were exported to SPSS version 27 for analysis. After cleaning for missing values, outliers, and inconsistencies, descriptive statistics summarized demographic and clinical characteristics, with findings presented in tables, graphs, and text. Annual changes in MDR-TB cases (2015–2023) were expressed as percentage change, calculated as [(final value – initial value)/|initial value|] × 100. Monthly cases distributions were also analyzed to assess seasonal patterns.

#### Time series analysis

Time series MDR/RR-TB data was broken down to look at its trend, seasonality, and randomness by time series decomposition approach [21],Moreover, the seasonal index (SI) was calculated to verify seasonality [22, 23]. A Seasonal ARIMA (SARIMA) model, incorporating seasonal autoregressive (SAR), differencing (SD), and moving average (SMA) components with a 12-month period (s = 12), was applied to capture recurring monthly patterns and forecast future cases [24, 25].

Data from January 2015 to December 2022 were used for model training, and 2023 data served as the test set. Analysis proceeded stepwise: [1] visual inspection of plotted patterns of the time series [2], stationarity testing using the augmented Dickey–Fuller (ADF) test [3], identification of autoregressive (AR) and moving average (MA) orders via autocorrelation function (ACF) and partial autocorrelation function (PACF) plots, and [4] model adequacy testing with the Ljung–Box Q test. Candidate models were compared using Akaike (AIC) and Bayesian (BIC) information criteria, with forecast accuracy validated by comparing predicted values against the 2023 test set. The final model generated forecasts for 2024–2025 and extended projections for the next decade.

All data preparation, model fitting, and diagnostics were conducted in R using specialized packages: tsibble for data handling, fable for SARIMA modeling and forecasts, tseries for the Augmented Dickey-Fuller and Ljung-Box tests, and ggplot2 for creating visualizations [26, 27].

### Ethical considerations

Ethical clearance was obtained from the Ethical Review Committee of Wollo University, College of Medicine and Health Sciences Wollo Tertiary Care and Teaching Hospital Tertiary Care Campus ensuring strict adherence to ethical guidelines and standards.

## Results

### Descriptive analysis

#### Sociodemographic characteristics

A total of 409 multidrug-resistant or rifampicin-resistant tuberculosis (MDR/RR-TB) cases were identified in the region during the study period (2015–2023). Thirty-seven cases were excluded due to inter-facility transfers after treatment initiation, resulting in a final analytical cohort of 372 cases. The annual case count showed a fluctuating upward trend, with the largest annual percentage increases observed between 2017 and 2019 (0% to 63.6%) and 2021–2022 (− 6.0% to 65.5%). Overall, 59.7% of patients were male, corresponding to a male-to-female ratio of 1.48 (Table 1).

**Table 1:** Demographic profile of MDR/RR-TB cases in Northeast Ethiopia

Variables		2015	2016	2017	2018	2019	2020	2021	2022	2023	Total
Percentage Change			−31.25	0	9	63.6	42.6	−6	65.5	25	
Sex	Female	16(33.3)	14(42.4)	15(45.5)	21(58.3)	25(46.3)	7(22.6)	10(34.5)	20(41.7)	22(36.7)	150(40.3)
	Male	32(66.7)	19(57.6)	18(54.5)	15(41.7)	29(53.7)	24(77.4)	19(65.5)	28(58.3)	38(63.3)	222(59.7)
Age	< 25	14(29.2)	10(30.3)	12(36.4)	9(25)	6(11.1)	8(25.8)	3(10.3)	8(16.7)	13(21.7)	83(22.3)
	25–65	34(70.8)	23(69.7)	21(63.6)	27(75)	46(85.2)	22(71)	25(86.2)	39(81.3)	47(78.3)	284(76.3)
	> 65	0	0	0	0	2(3.7)	1(3.2)	1(3.4)	1(2.1)	0	5(1.3)
Total MDR/RR-TB Cases		48	33	33	36	54	31	29	48	60	372

This table presents the annual distribution of multidrug-resistant/rifampicin-resistant tuberculosis (MDR/RR-TB) cases by sex and age group. Values are expressed as number (percentage) of cases within each category. The row “Percentage Change” indicates the year-to-year percentage change in total MDR/RR-TB cases compared with the preceding year

**Fig. 2:**
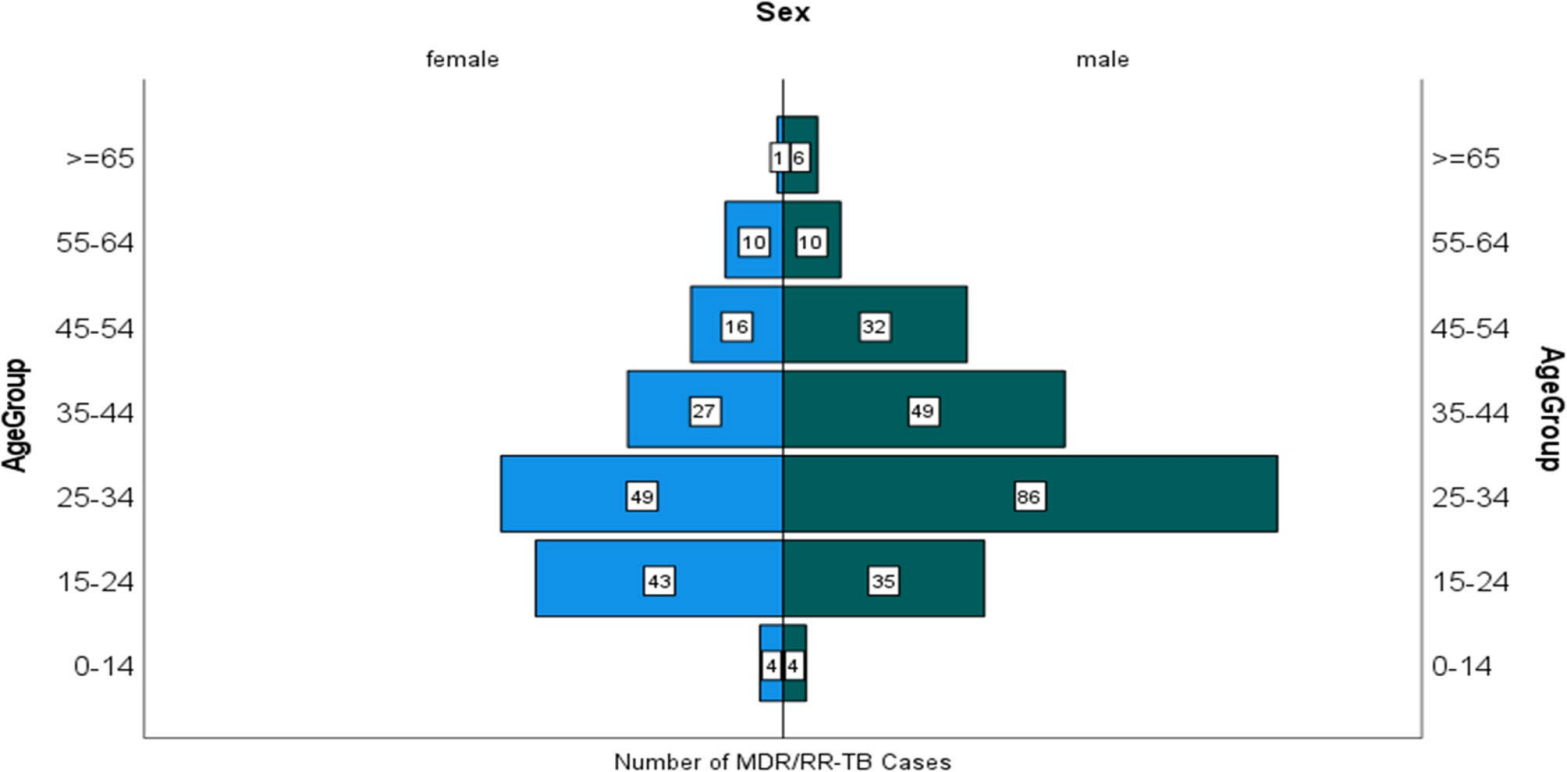
MDR/RR-TB cases by age and gender from 2015 to 2023

MDR/RR-TB cases were distributed across all age groups but were highest among young and middle-aged adults (15–64 years). Within this demographic, males accounted for a majority of cases (56.9%, *n* = 212), compared to females (38.9%, *n* = 145). Pediatric cases (age < 15 years) represented 2.1% (*n* = 8) of the total cohort (Fig. 2).

#### Clinical characteristics of MDR/RR-TB of Northeast Ethiopia

A majority of cases (55.4%) had a history of previous TB treatment. Disease manifestation was predominantly pulmonary (91.1% of cases). Nearly all patients (97.7%) were diagnosed via the GeneXpert platform. In contrast, only a single case (0.3%) reported a known history of contact with an MDR/RR-TB patient (Table 2).

**Table 2:** Clinical characteristics of MDR/RR-TB cases in Northeast Ethiopia 2015–2023

Variables		2015	2016	2017	2018	2019	2020	2021	2022	2023	Total
Resistant Type	MDR	9	4	7	0	6	0	2	2	17	47(12.6)
	RR	39	29	26	36	48	31	27	46	43	325(87.4)
Previous TB treatment	Firstline Anti-TB Drugs	32(8.6)	15(4.0)	10(2.7)	11(3.0)	21(5.6)	13(3.5)	10(2.7)	21(5.6)	30(8.1)	163(43.8)
	Second line Anti-TB Drug	1(0.3)	1(0.3)	0	0	2(0.5)	1(0.3)	2(0.5)	4(1.1)	4(1.1)	15(4.0)
	Both first- and second-line anti-TB	6(1.6)	6(1.6)	5(1.3)	1(0.3)	1(0.3)	0	1(0.3)	4(1.1)	4(1.1)	28(7.5)
	No	9(2.4)	11(3.0)	18(4.8)	24(6.5)	30(8.1)	17(4.6)	16(4.3)	19(5.1)	22(5.9)	166(4(1)4.6)
Site of Disease	Extra pulmonary	5(10.4)	4(12.1)	2(6.1)	3(8.3)	3(5.6)	3(9.7)	0	6(12.5)	7(11.7)	33(8.9)
	Pulmonary	43(89.6)	29(87.9)	31(93.9)	33(91.7)	51(94.4)	28(90.3)	29	42(87.5)	53(88.3)	339(91.1)
Diagnosed Using	LPA	2(4.2)	0	0	0	0	0	0	0	0	2(0.5)
	Other	0	0	0	0	1(1.9)	0	0	0	4(6.7)	5(1.3)
	Pheno typic DST	0	0	0	0	0	0	0	3(6.3)	0	3(0.8)
	Gene X-pert	46(95.8)	33	33	36	53(98.1)	31	29	45(93.8)	56(93.3)	362(97.3)
HIV status	Negative	38(79.2)	24(72.7)	27(81.8)	29(80.6)	31(57.4)	25(80.6)	26(89.7)	40(83.3)	54(90)	294(79)
	Positive	10(20.8)	9(27.3)	6(18.2)	7(19.4)	23(42.6)	6(19.4)	3(10.3)	8(16.7)	6(10)	78(21)
Contact History with Known MDR/RR-TB Patients	No	48	32(97)	33	36	54	31	29	48	60	371(99.7)
	Yes	0	1(3)	0	0	0	0	0	0	0	1(0.3)
Nutritional Status	MAM	6(12.5)	1(3)	5(15.2)	2(5.6)	18(33.3)	10(32.3)	6(20.7)	9(18.8)	11(18.3)	68(18.3)
	Normal	29(60.4)	26(78.8)	23(69.7)	25(69.4)	25(46.3)	14(45.2)	17(58.6)	27(56.3)	28(46.7)	214(57.5)
	SAM	13(27.1)	6(18.2)	5(15.2)	9(25)	11(20.4)	7(22.6)	6(20.7)	12(25)	21(35)	90(24.2)
Eligible For regimen	Individualized Long	1				1				3	5(1.3)
	Long	47(12.6)	33(8.8)	33(8.8)	29(7.8)	26(7)	23(6.3)	17(4.6)	35(9.4)	31(8.3)	261(73.7)
	Short	0	0	0	7(1.9)	27(7.3)	8(2.2)	12(3.2)	13(3.5)	26(7)	106(25)

This table summarizes the clinical profiles of multidrug-resistant/rifampicin-resistant tuberculosis (MDR/RR-TB) cases over a nine-year period. Variables include drug resistance type, history of previous TB treatment, site of disease, diagnostic method, HIV status, history of contact with known MDR/RR-TB patients, nutritional status, and treatment regimen eligibility. Values are presented as number (percentage) of cases within each category for the respective year

Abbreviations:* MDR* multidrug-resistant, *RR* rifampicin-resistant, *TB* tuberculosis, *LPA* line probe assay, *DST* drug susceptibility testing, *MAM* moderate acute malnutrition, *SAM* severe acute malnutrition

**Fig. 3:**
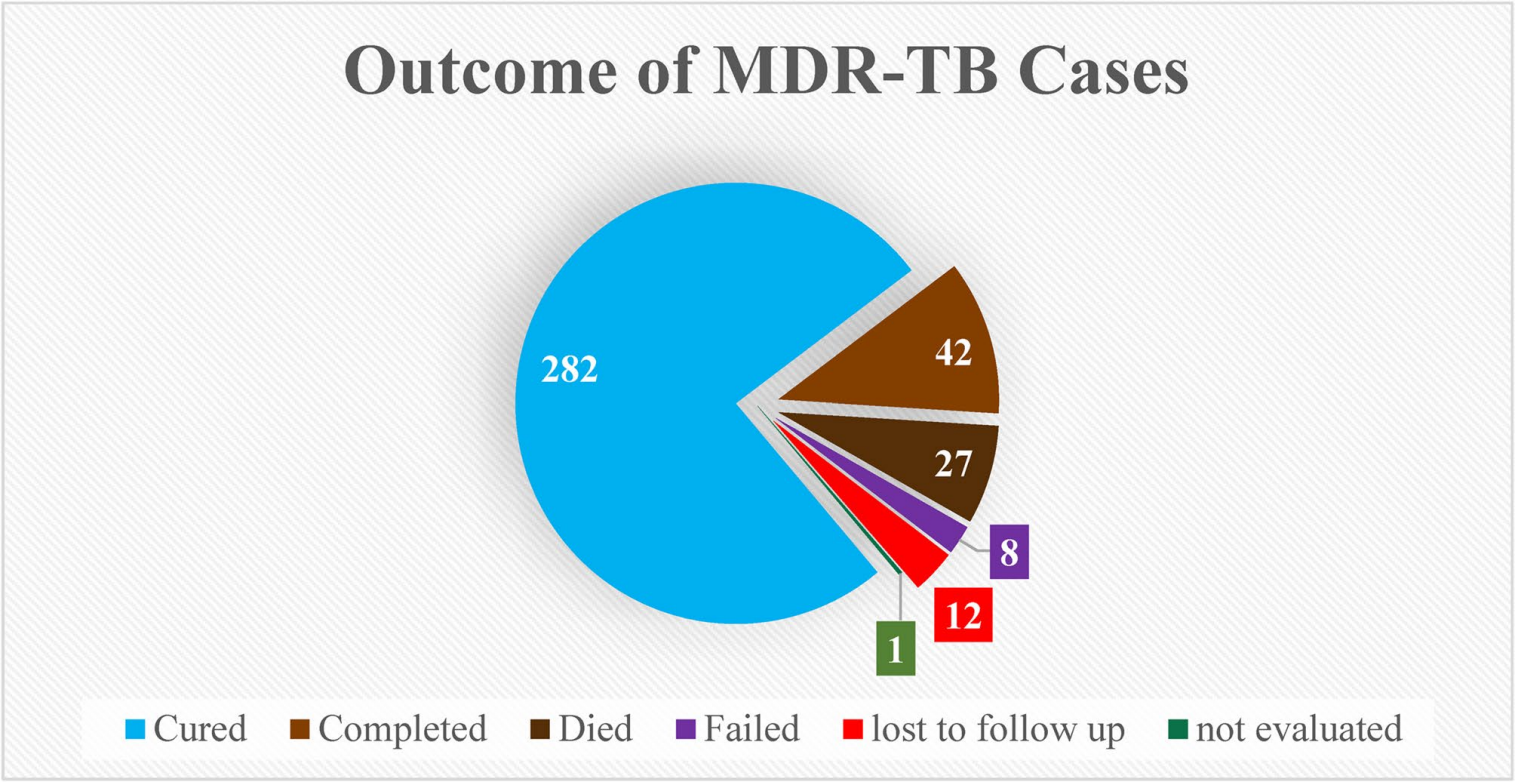
Magnitude of treatment outcome of MDR/RR-TB patients at six TICs Northeast Ethiopia from 2015 to 2023

The overall prevalence of unfavorable treatment outcomes was 7.3% (*n* = 27) for death and 2.2% (*n* = 8) for treatment failure. As illustrated in Fig. 3, a majority of patients 87.0%(*n* = 309) experienced a favorable outcome, defined as either cure or treatment completion. Twelve patients (3.2%) were lost to follow-up, resulting in an unknown outcome status. A history of first-line treatment was reported by the majority of cases, whereas a history of both first- and second-line anti-TB drug treatment was reported by 7.5%.

#### Time-series analysis

**Fig. 4:**
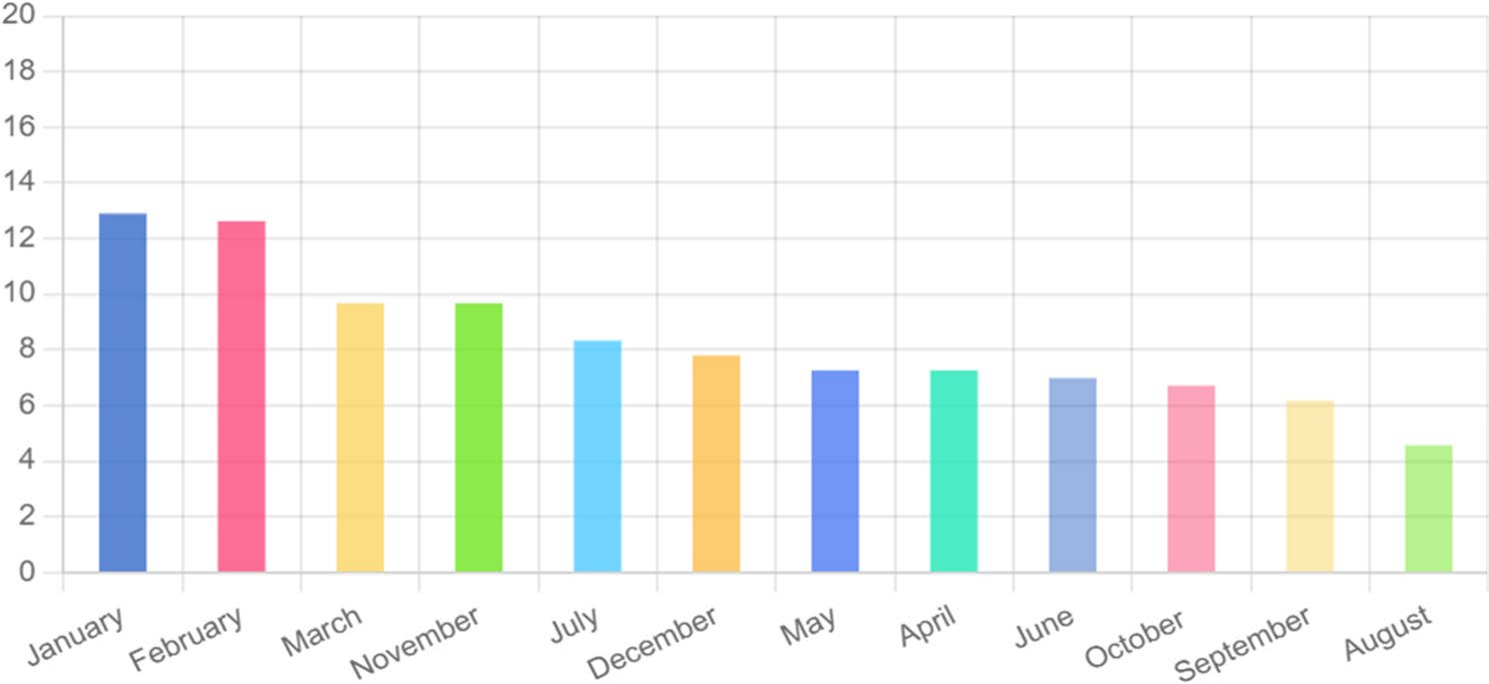
Percentage of month of occurrence of MDR/RR-TB from 2015-2023

Analysis of the seasonal indices (Table 3) confirmed a clear seasonal pattern in the incidence of MDR/RR-TB cases in Northeast Ethiopia. The seasonal trough typically occurred in August. Following this low point, case numbers demonstrated a general upward trend from August through November, after which they began to decline. This pattern is visually summarized in Fig. 4, which presents the monthly percentage distribution of MDR/RR-TB cases from 2015 to 2023.

**Table 3:** The Seasonal Index (SI) of MDR-TB cases distribution in months from 2015 to 2023 in Northeast Ethiopia

Months	2015	2016	2017	2018	2019	2020	2021	2022	2023	Total	Average number of cases per month	Monthly Average	SI
January	7	5	1	10	8	2	4	2	9	48	5.33	3.482	1.53
February	6	6	7	1	9	6	4	3	5	47	5.22	3.482	1.5
March	5	3	4	0	3	6	1	7	7	36	4	3.482	1.2
April	4	2	1	4	4	0	5	4	3	27	3	3.482	0.86
May	3	4	2	1	4	0	5	4	4	27	3	3.482	0.86
June	3	1	3	3	1	2	4	5	4	26	2.89	3.482	0.83
July	6	2	4	2	5	2	1	0	9	31	3.44	3.482	1
August	1	0	1	1	4	1	3	3	3	17	1.88	3.482	0.54
September	1	1	2	1	3	4	1	8	2	23	2.55	3.482	0.74
October	4	4	1	2	5	3	0	3	3	25	2.77	3.482	0.8
November	4	3	3	7	5	3	1	7	3	36	4	3.482	1.2
December	4	2	4	4	3	2	0	2	8	29	3.22	3.482	0.93

This table shows the monthly distribution of multidrug-resistant tuberculosis (MDR-TB) cases over nine years, including the average number of cases per month, monthly average, and calculated seasonal index (SI)

The identification of a distinct seasonal pattern in the data justified the use of a Seasonal Autoregressive Integrated Moving Average (SARIMA) model, formally denoted as SARIMA(p, d, q) × (P, D, Q)m. In this notation, (p, d, q) represent the order of the non-seasonal autoregressive, integrative, and moving average components, respectively; (P, D, Q)s signify their seasonal counterparts; and *m* defines the period of the seasonality.

A seasonal index of 1.53 for January indicates MDR-TB cases are about 53% higher than the overall monthly average, showing seasonality, whereas an index of 0.54 in August reflects 46% fewer cases than average.

**Fig. 5:**
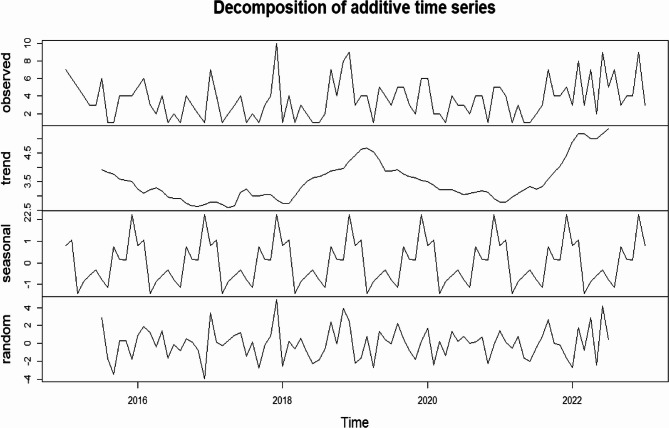
Time series decomposition of MDR-TB cases from January 2015 to December 2023

Time series decomposition of the MDR/RR-TB case data revealed the underlying trend, seasonal, and irregular (random) components (Fig. 5).

Prior to model parameterization, the stationarity of the time series was formally assessed using the Augmented Dickey-Fuller (ADF) test. A non-significant result from this test indicates a non-stationary series, which must be corrected to avoid spurious regression and to ensure reliable parameter estimation in the SARIMA model.

The initial ADF test on the raw data yielded a test statistic of *t* = −4.3285 (*p* = 0.01), which was significant at the α = 0.05 level, suggesting the series was stationary. However, to rigorously account for the identified strong seasonality and ensure optimal model performance, first-order non-seasonal differencing (d = 1) and first-order seasonal differencing (D = 1) were applied. After applying these transformations, the ADF test result strengthened considerably (*t* = −5.798, *p* < 0.01), providing robust evidence of an effectively stationary series.

Following the establishment of stationarity, the Autocorrelation Function (ACF) and Partial Autocorrelation Function (PACF) plots for the differenced series were examined to identify potential values for the SARIMA model parameters (p, d, q) × (P, D, Q)m.

The auto.arima function was used to generate 12 candidate SARIMA models, which were subsequently compared to identify the best-fitting model. Based on an evaluation of goodness-of-fit statistics, the SARIMA(0,0,0)(0,0,1)[12] model was selected as optimal, as it yielded the lowest values for both the Akaike Information Criterion (AIC) and the Bayesian Information Criterion (BIC). Furthermore, all parameter estimates were statistically significant, confirming the model’s adequacy (Table 4).

**Table 4:** Comparison among proposed SARIMA model candidates applied for MDR/RR-TB forecasting from 2015 to 2023 in Northeast Ethiopia

Model	Estimate	T	Stder	*P*	Ljung-Box Q Test		AIC	BIC	RMSE	MAPE
					Statistics	*P*-value				
SARIMA(0,0,0)(0,0,1)_12_	3.732	14.35	0.260	0.000	10.425	0.404	426.1018	433.8259	2.1023	75.584
SMA1	0.2341	0.1298	1.8032	0.071						
SARIMA(0,0,0)(0,0,1)_12_ zero mean	0.907	2.966358	0.3058	0.0030	22.964	0.0108	507.016	512.1449	3.01	72.223
SARIMA(0,0,0)(1,0,1)_12_	3.719	15.25	0.2438	0.000	11.42	0.325	420.244	430.502	1.937	70.414
SAR1	−0.6132	−4.777	0.1283	1.778						
SMA1	0.999	6.1172	0.1634	9.522						
ARIMA(0,0,0)	3.6770	0.2202	16.6965	0	9.4847	0.4868	424.102	429.2309	2.157	78.493
SARIMA(0,0,1)(0,0,1)_12_	3.741	13.523	0.276	0.000	10.076	0.4339	424.1047	434.3621	2.1068	75.325
MA1	0.0705	0.7863	0.08967	0.4316						
SMA1	0.2185	1.6689	0.1309	0.0951						
SARIMA(1,0,0)(0,0,1)_12_	3.744	13.135	0.285	0.0000	10.038	0.437	423.870	434.128	2.10451	75.156
AR1	0.0981	0.9188	0.1068	0.3581						
SMA1	0.2136	1.630	0.1310	0.1029						
SARIMA(0,0,0)(1,0,0)_12_	3.7349	14.1144	0.264	0.0000	10.078	0.4336	423.0294	430.722	2.118	75.990
SAR1	0.1988	1.780	0.111	0.0749						
ARIMA(0,0,0)(2,0,0)_12_	3.7328	14.398	0.2592	0.00000	10.178	0.425	424.957	435.214	2.11704	76.025
SAR1	0.20695	1.7819	0.1161	0.07476						
SAR2	−0.03199	−0.2692	0.1188	0.7877						
SARIMA(0,0,0)(0,0,2)_12_	3.73408	14.8736	0.2510	0.00000	11.118	0.0000	424.256	434.514	0.3484	75.508
SMA1	0.2682	1.8504	0.1449	0.06425						
SMA2	−0.0991	−0.6472	0.1531	0.5174						
ARIMA(0,0,0)(1,0,2)_12_	3.7244	14.955	0.2490	0.00000	11.379	0.328	422.188	435.0097	1.9429	70.553
SAR1	−0.6488	−3.35885	0.1931	7.82670						
SMA1	1.04052	4.5334	0.2295	5.8015						
SMA2	0.0405	0.2385	0.1699	8.114						
SARIMA(1,0,0)(1,0,0)_12_	3.740	13.038	0.2868	0.0000	9.599	0.4763	424.169	434.426	2.1094	75.521
AR1	0.0992	0.929	0.10674	0.35268						
SAR1	0.1825	1.60193	0.1139	0.1091						
SARIMA(1,0,1)(0,0,1)_12_	3.8026	9.5936	0.3963	0.0000	9.059	0.5264	424.496	437.318	2.088	74.400
AR1	0.7988	2.2547	0.3543	0.0241						
MA1	−0.699	−1.677	0.4171	0.0934						
SMA1	0.2181	1.7276	0.1262	0.0840						
SARIMA(2,0,2)(1,0,1)_12_	3.7506	13.293	0.2821	0.0000	4.399	0.9275	420.600	441.115	1.840	65.8227
AR1	−1.00199	−2.8645	0.3497	4.1760						
AR2	−0.2718	−0.80097	0.3394	4.2314						
MA1	1.1193	3.5738	0.313	3.5180						
MA2	0.5276	1.7937	0.2941	7.28586						
SAR1	−0.5369	−4.0437	0.1327	5.2591						
SMA1	0.9999	0.1788	5.5907	2.2605						
ARIMA(0,0,0)_12,0_ mean	0.26007				9.4847	0.4868	552.851	555.416	4.2634	100
SMA1	0.1298									

*AIC* Akaike information criterion, *BIC* bayesian information criterion, *RMSE* root mean square error, *MAPE* mean absolute percent error

The effect of the SARIMA (0,0,0)(0,0,1)_12_ model was evaluated by comparing its predicted values against the actual data recorded from January to December 2023.

**Table 5:** Comparison of actual values and predicted values from January to December 2023

Month	Actual Values	Prediced Values	95% CI	
			LCI	UCI
January	9	4	0	8
February	5	5	1	9
March	7	4	0	8
April	3	5	0	9
May	4	3	0	7
June	4	5	1	9
July	9	4	0	8
August	3	5	0	9
September	2	3	0	7
October	3	4	0	8
November	3	4	0	8
December	8	5	1	9

Comparison of observed (actual) values and model-predicted values with corresponding 95% confidence intervals (CI) from January to December 2023. LCI = lower confidence interval; UCI = upper confidence interval. In most months, the actual values were within or close to the predicted 95% CI range, indicating reasonable model performance, although some deviations (e.g., January and July)

With the exception of the actual values in January and July, all other observed data points fell within the 95% confidence interval (CI) of the predicted values. Furthermore, the actual trend from 2018 to 2020 closely matched the predicted trend, demonstrating the model’s reliability (Fig. 7).

The SARIMA model forecasts 44 (95% CI: 0–96) and 45 (95% CI: 0–96) new MDR/RR-TB cases for Northeast Ethiopia in 2024 and 2025, respectively.

The forecast predicts stability in the annual number of new MDR/RR-TB cases over the next decade, averaging approximately 45 cases per year.

## Discussion

Descriptive analysis showed that MDR/RR-TB mainly affected young to middle-aged men, consistent with WHO and national reports, highlighting potential economic losses. Most cases (55.4%) were acquired MDR-TB, linked to prior TB treatment, aligning with Ethiopian studies citing poor adherence, inappropriate prescribing, and pharmacokinetic factors [28–30].

Our study period overlapped with the COVID-19 pandemic, during which disruptions to TB services, reduced access, diagnostic delays, and treatment interruptions likely contributed to variability in MDR/RR-TB cases [31, 32], consistent with evidence on the broader health system burdens caused by COVID-19 and Long COVID [33]. Cancer treatment can also influence drug resistance, as chemotherapy and immunosuppressive therapy increase TB reactivation risk and hinder adherence, underscoring the need for integrated management of comorbidities [34].

A sharp decline in case notifications observed in 2020 is attributable to the synergistic effects of the pandemic (e.g., diverted healthcare resources, lockdowns) and regional conflict, which severely limited healthcare access [35]. The subsequent surge in notifications in 2022 likely represents a combination of the detection of previously undiagnosed cases and the successful transition to a more accessible ambulatory care model [36].

A clear seasonal pattern was identified, with case counts consistently rising during the winter dry season (Bega). This trend may be linked to increased social interactions at post-harvest events such as religious festivals, weddings, and trade activities, which create crowded conditions favorable for TB transmission [37]. The consistent trough in cases each August further supports the role of socio-cultural and seasonal factors in disease dynamics.

**Fig. 6:**
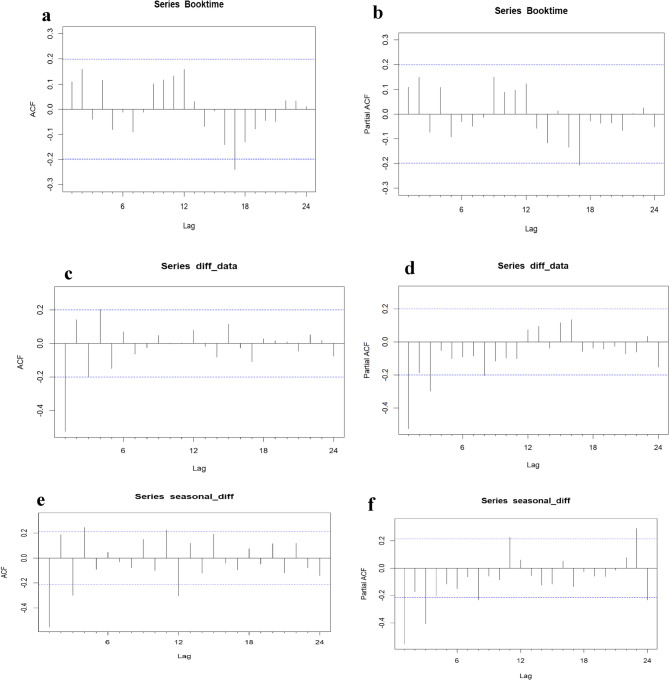
The ACF and PACF plots for estimating the stationary or lags. **a** The ACF graph of the raw data (d = 0 and D = 0); **b** the PACF graph of the raw data (d = 0 and D = 0); **c** the ACF graph of one-order trend difference data (d = 1 and D = 0); **d** the PACF graph of one-order trend difference data (d = 1 and D = 0); **e** the ACF graph of one-order seasonal difference data (d = 1 and D = 1); **f** the PACF graph of one-order seasonal difference data (d = 1 and D = 1)

**Fig. 7:**
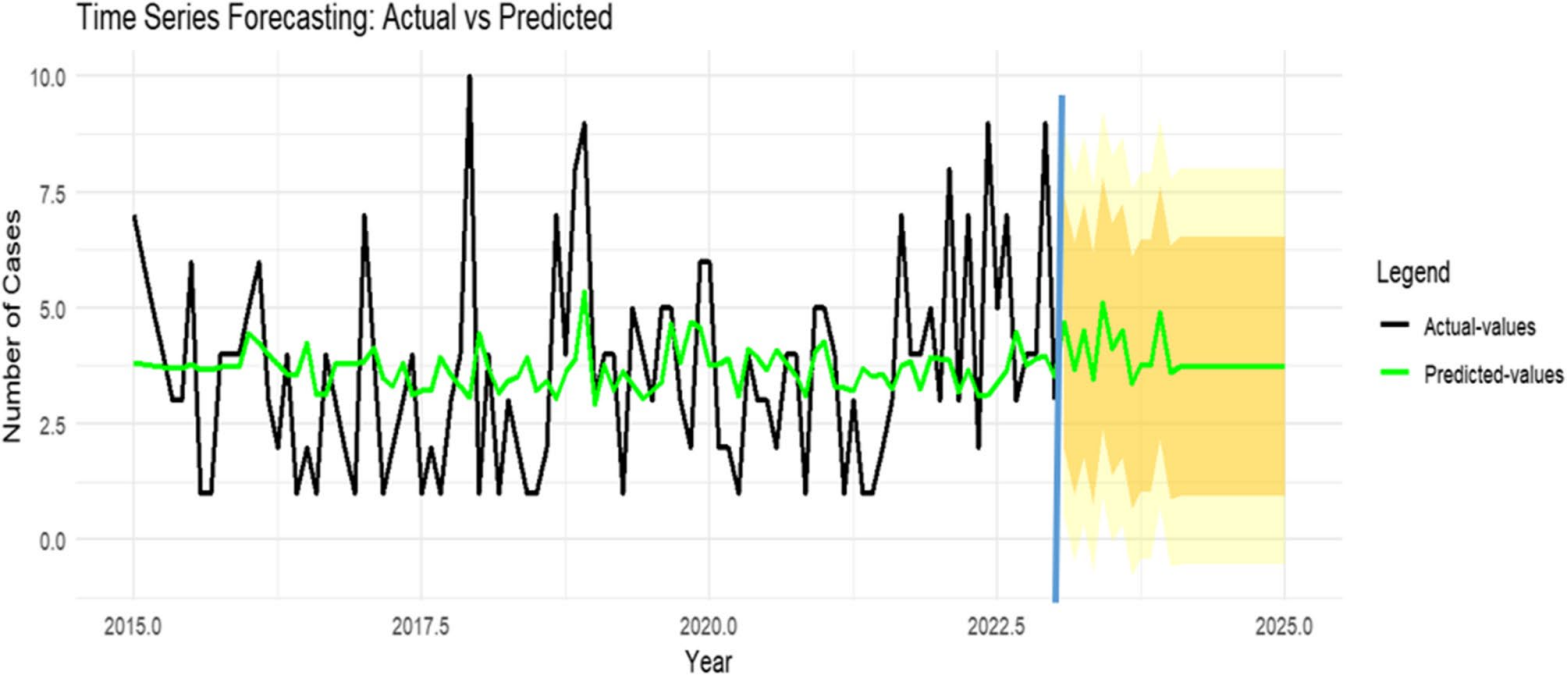
Comparison of actual and predicted cases of MDR-TB in Northeast Ethiopia. The black and green light represent the observed and predicted values respectively; after blue vertical line, the orange and yellow represent 80 % and 95 % confidence interval, respectively

**Fig. 8:**
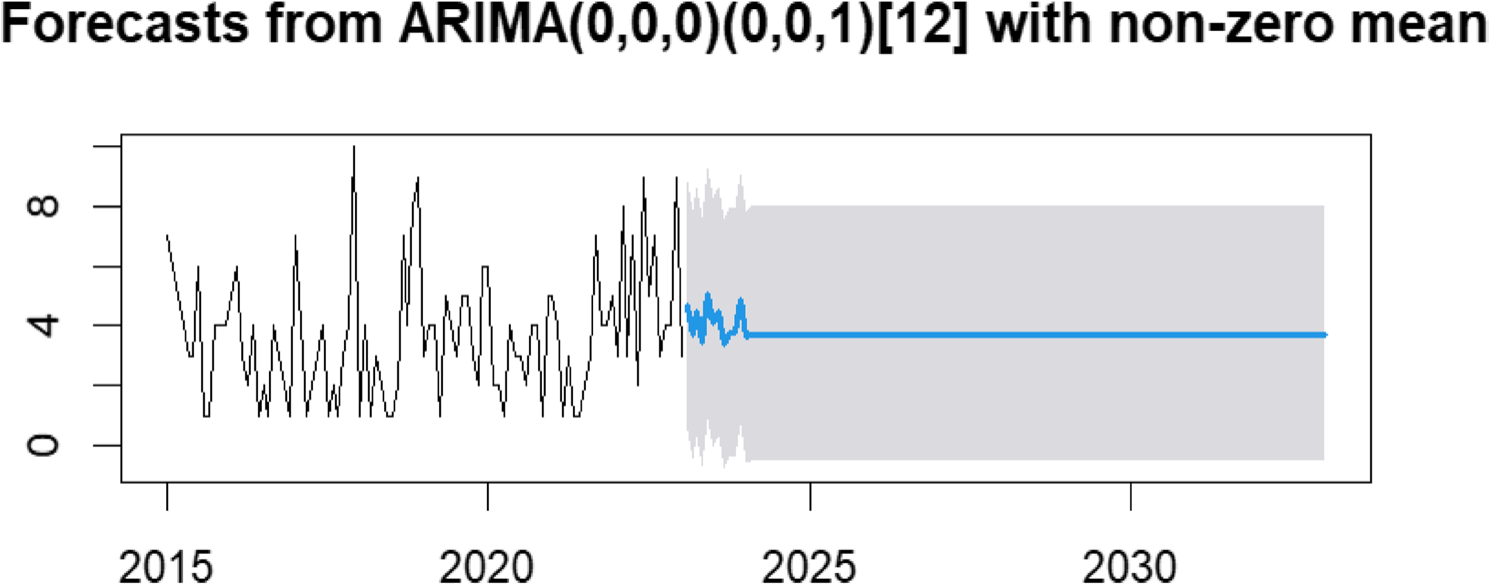
Forecast of multidrug-resistant/rifampicin-resistant tuberculosis (MDR/RR-TB) cases in Northeast Ethiopia, 2025–2035, using SARIMA (0,0,0)(0,0,1) [12] model.The figure presents the time series forecast of MDR/RR-TB cases over a 10-year period. The solid line indicates the observed data, while the blue shaded area represents the forecast with 95% confidence intervals. The model projects a stable trend at an average of ~ 45 cases per year

Annual case counts were compiled into a time series and analyzed in R. Visual inspection and autocorrelation function (ACF) plots (Fig. 6) confirmed the presence of both trend and seasonal components. Stationarity was achieved through first-order non-seasonal and seasonal differencing, confirming the suitability of a SARIMA modeling approach. The selected SARIMA(0,0,0)(0,0,1)[12] model, chosen for its minimization of Akaike (AIC) and Bayesian (BIC) Information Criteria, demonstrated a strong fit. Forecasted values closely aligned with observed data, with most points within the 80% and 95% confidence intervals (Fig. 7). The model accurately captured the 2022 peak, reflecting the impact of programmatic changes and conflict-related disruptions. The 10-year forecast projects a stable trend of approximately 45 cases per year (Fig. 8). Projected stability in MDR/RR-TB incidence reflects restored case detection post-disruption but indicates persistent transmission and insufficient control measures to reduce burden [38].

The SARIMA model relies only on historical case data and does not account for external factors such as socioeconomic conditions or environmental influences. Consequently, forecasts are associated with wide confidence intervals and are more reliable for short-term prediction; long-term projections require regular updating with new data to maintain validity. The regional focus and limited sample size also constrain generalizability, highlighting the need for nationwide studies. In conclusion, our findings suggest that current interventions may not be sufficient to substantially reduce the MDR/RR-TB burden in the coming decade, potentially hindering progress toward the WHO End TB Strategy targets.

## Conclusions

This study is first to examine the temporal patterns and trends of MDR/RR-TB in Northeast Ethiopia. The conflict between the government forces and Rebel group likely contributed to delays in treatment, persisting until the fighting ceased in November 2021. As a result, a notable increase in hospital admission was recorded in January. Seasonal variations were also observed, with the highest case numbers occurring during the Bega(winter) season and lowest in August. This result highlights the importance of ongoing surveillance among MDR/RR-TB. Control strategies of MDR/RR-TB should target high-risk periods particularly during the Bega (winter) season, and targeted resource allocation could improve case detection and expand timely treatment access.

## Data Availability

The datasets generated and analyzed during the current study are not publicly available due to the fact that it contains personal information, but are available from the corresponding author on reasonable request.

## Abbreviations

ACF: Autocorrelation Function
AIDS: Acquired Immuno-Deficiency Syndrome
AIC: Akaike information criterion
ARIMA: Autoregressive Integrated Moving Average
SARIMA: Seasonal Autoregressive Integrated Moving Average
BIC: Bayesian information criterion
DR-TB: Drug Resistant TB
DST: Drug Susceptibility Testing
MDR-TB: Multidrug-Resistant Tuberculosis
MDR/RR-TB: Multidrug/rifampicin-resistant tuberculosis
MAPE: Mean Absolute Percent Error
PACF: Partial Autocorrelation Function
RMSE: Root Mean Square Error
RR-TB: Rifampicin Resistant TB
TB: Tuberculosis
TIC: Treatment Initiation Center
